# Establishment of a Mandarin Chinese Version of the Oral Frailty Index-8 and Exploration of the Association Between Oral Frailty and Sarcopenia

**DOI:** 10.3390/geriatrics10020047

**Published:** 2025-03-17

**Authors:** Chen-Cheng Yang, Hsiang-Tai Chen, Katsuya Iijima, Tomoki Tanaka, Chia-Yen Dai, Sang-Ju Yu, Hung-Yi Chuang

**Affiliations:** 1 Department of Occupational Medicine, Kaohsiung Municipal Siaogang Hospital, Kaohsiung Medical University, Kaohsiung 812, Taiwan; abcmacoto@gmail.com; 2 Department of Occupational and Environmental Medicine, Kaohsiung Medical University Hospital, Kaohsiung Medical University, Kaohsiung 812, Taiwan; daichiayen@gmail.com; 3 Taiwan Society of Home Health Care, Taipei 100, Taiwan; 4 College of Health and Medicine, University of Tasmania, Hobart, TAS 7001, Australia; 5 Institute of Gerontology, the University of Tokyo, Tokyo 113-8654, Japan; iijima@iog.u-tokyo.ac.jp (K.I.);; 6 Home Clinic Dulan, Taitung 959, Taiwan; 7 Department of Public Health and Environmental Medicine, College of Medicine, Kaohsiung Medical University, Kaohsiung 812, Taiwan

**Keywords:** oral frailty, sarcopenia, Oral Frailty Index-8, SARC-F, frailty, questionnaire

## Abstract

**Objective:** The aim of our study was to introduce a Mandarin Chinese version of the oral frailty assessment and explore the relationship between oral frailty and sarcopenia. A total of 409 elders (171 male, 238 female) participated in surveys using the Mandarin Chinese version of the Oral Frailty Index-8 (OFI-8) in Kaohsiung, Taiwan. **Method:** The translation of the Mandarin Chinese version of OFI-8 adhered to the Consensus-based Standards for the Selection of Health Measurement Instruments (COSMIN) reporting guidelines. The eight-item questionnaire assessed tooth status, oral function, and other subjective measures. Additionally, sarcopenia was evaluated using the SARC-F questionnaire. **Result:** Among the participants, 195 participants were classified as non-oral frailty and 214 participants were oral frailty. Significant differences were observed in age, gender, body mass index (BMI), education level, and scores on the SARC-F questionnaire between the non-oral frailty and oral frailty populations. In logistic regression model, oral frailty showed a significant and positive association with the SARC-F score (adjusted odds ratio 2.130, 95% confidence interval 1.580–2.872, *p*-value < 0.001), even after adjusting for age, gender, BMI, and education level. **Conclusion:** This study has developed a valuable Mandarin Chinese version of the oral frailty screening questionnaire, the OFI-8. Oral frailty is significantly and positively associated with a higher risk of sarcopenia, particularly among the elderly, males, and those with lower education levels. This measure proves to be practical for assessing oral health status in the Chinese community, promoting oral frailty research within the Mandarin Chinese population, and addressing the challenges associated with defining oral frailty in future studies.

## 1. Introduction

With recent medical advancements worldwide, the average life expectancy of individuals is increasing, leading to a global rise in aging populations. This shift is evident in countries like Taiwan, which transitioned from an aging society to an aged society in 2018, when more than 14% of the national population was aged 65 years and older [1]. The heightened prevalence of the aged population corresponds with an increased incidence of diseases attributed to the frailty of bodily organs. In medical terms, frailty is defined as a condition in which patients experience a decline in physiological reserve and an elevated vulnerability to stressors, often associated with the aging process [2]. Various forms of research have been undertaken to study biopsychosocial frailty for the improvement of human health, and within this context, sarcopenia is recognized as a key indicator of physiological frailty. Sarcopenia is a type of muscle atrophy that contributes to the involuntary loss of skeletal muscle mass and overall body strength. However, a similar severity of symptoms appearing in oral health seems to be more neglected in the current research field. Oral frailty or poor oral health, as another form of degeneration of bodily organs among the elderly population, seems to have attracted less attention in the field. To date, many studies have researched the different aspects of sarcopenia disease, and a few studies have suggested that sarcopenia can originate through oral frailty via a deleterious diet; thus, poor oral health shows an adverse influence on sarcopenia patients [2,3]. Poor oral health may restrict food selection, which may affect nutrition intake, leading to malnutrition and oral diseases such as periodontal disease; chronically, it will lead to sarcopenia, which affects the patient’s muscle movement relating to swallowing, jaw opening, and lip function, further restricting their food intake and nutrition consumption [2,4,5]. Due to the inconsistency in the definition of oral frailty and its multi-faceted factors, as well as the highly overlapping definitions of oral health, this finding is rather unclear in the field and warrants further investigation. Oral frailty is defined as a decrease in oral functions due to age; it is assessed from a multi-item perspective, including tooth loss, oral hygiene, oral diseases, and chewing and swallowing muscle function [2,3]. Sarcopenia disease is a decrease in psychological function which also includes the loss of swallowing muscles. By considering the symptoms of sarcopenia, studies focusing on oral frailty will provide a more comprehensive understanding of sarcopenia. To date, sarcopenia studies that focus on the field of oral health have been rather limited; however, the need to understand sarcopenia symptoms is vital, as sarcopenia drastically increases the mortality rate among the elderly population [6].

Sarcopenia is associated with a higher rate of fall incidence, mortality, and functional decline [7]. Sarcopenia may not be an acute cause of death, but it may instead result in frailty. This has given rise to the field of sarcopenia research in the current literature. There are several methods of screening for or diagnosing sarcopenia; these include the finger-ring test to test grip strength, the chair stand test to test the functional fitness level, and the gait speed test for examine mobility as well as functional capacity. All these are screening tools for sarcopenia symptoms [8,9]. Other easily self-reported assessments, such as the SARC-F questionnaire, have also been introduced to screen for sarcopenia symptoms [10]. The SARC-F questionnaire focuses on five different physical function questions regarding the participant’s strength, assistance with walking, rising from a chair, difficulties in climbing stairs, and fall experiences [10]. In the clinical field, formal diagnostic methods required results coming from dual-energy X-ray absorptiometry (DXA), bioelectrical impedance assessments, computed tomography, magnetic resonance imaging, or ultrasound can be used to measure body composition [11].

The prevalence of sarcopenia is notably high among the elderly population. A 2019 study from Sweden involving 287 elderly participants aged 85 to 89 years old revealed that 21% of the population had sarcopenia [12]. Subsequent statistics from the follow-up period indicated that individuals with sarcopenia had a higher mortality rate compared to participants without sarcopenia. The study argued that sarcopenia is not only associated with a negative impact on daily activity, mobility, and quality of life but also has a detrimental effect on metabolic regulation, potentially leading to a higher prevalence of diabetes [12]. This serves as a stark illustration of the importance of sarcopenia research in the context of aging societies internationally.

To date, studies have focused on several dimensions of sarcopenia research, such as muscle mass, muscle strength, and physical performance. However, there is a scarcity of research focusing on the association between oral health assessment and sarcopenia, and till now, this has still not been totally understood [13,14]. Furthermore, based on the current literature, no oral frailty screening tool has been developed in Taiwan, and no association between sarcopenia and oral frailty has been discussed. In 2021, a Japanese researcher made a questionnaire that specifically focused on oral health and the concept of oral frailty associated with sarcopenia [15]. The study showed some significant results in showing the relationship between oral frailty and sarcopenia. Due to the inconsistent results and relatively rare studies in this field, the purpose of our study was to establish a Mandarin Chinese version of the Oral Frailty Index-8 (OFI-8) questionnaire and to explore the association between oral frailty and sarcopenia.

## 2. Materials and Methods

### 2.1. Subjective

This cross-sectional study was reviewed and approved by the Kaohsiung Medical University Hospital Institutional Review Board [IRB number KMUHIRB-E(I)-20220048]. This study was conducted between April 2022 and July 2024. We recruited participants from residents of the community (Siaogang District, Qianzhen District, Fengshan District, Daliao District, and Linyuan District) near Kaohsiung Municipal Siaogang Hospital. The inclusion and exclusion criteria can be summarized as follows: This study included adult participants who were physically capable of independent walking and exhibited no signs of mental health conditions, including delirium or cognitive impairment. This study excluded participants with severe mobility impairments, cognitive deficits, or those unable to comprehend or complete the assessment. Participants provided written informed consent after receiving sufficient explanation prior to their participation in this study. This study recruited participants through community health promotion activities in Kaohsiung City, and all 409 participants who joined the activities were included in this study. All participants were asked to complete a survey on demographic characteristics that included age, gender, education level, and exercise habits, defined as engaging in physical activity more than three times a week for over 30 min per session, along with body mass index (BMI) measurements. All data were collected by well-trained interviewers.

### 2.2. Development of Mandarin Chinese Version of OFI-8 Questionnaire

The multiple dimensions of oral frailty were evaluated using the Mandarin Chinese version of the OFI-8 questionnaire, which consists of a total of 8 items rated on scales ranging from 0 to 1 or 0 to 2. The OFI-8 questionnaire serves as a screening tool for oral frailty and is constructed of eight different questions addressing various aspects of oral health. These aspects include the number of teeth, dentures, tooth hygiene, frequency of dentist visits, condition of swallowing muscles, biting force, and symptoms related to oral diseases. This study developed the Mandarin Chinese version of the questionnaire based on the International Society of Pharmacoeconomics and Outcomes Research (ISPOR) task force guidelines [16]. Initially, this study sought and obtained permission from Professor Katsuya Iijima (University of Tokyo), the original developer of the OFI-8 questionnaire, to translate the questionnaire into Mandarin Chinese. The translation process involved forward-translation followed by reconciliation, back-translation, back-translation review, harmonization, and cognitive debriefing. Two experts proficient in both Mandarin Chinese and Japanese who were unaware of this study’s objective conducted the back-translation. Through random sampling, seven Mandarin Chinese-speaking participants, including a community leader and healthcare professionals, were selected for the cognitive debriefing sessions. If any of the seven participants encountered difficulty understanding any sentence in the 8 items, they were invited to provide feedback, revise the wording, and contribute to the harmonization process. The results obtained in these different stages were synthesized to formulate the final measure. Please see Supplementary Table S1 for the complete Mandarin Chinese version of the OFI-8 questionnaire.

### 2.3. SARC-F Questionnaire

The SARC-F questionnaire serves as a screening tool specifically designed for sarcopenia symptoms, comprising 5 different elements to assess the risk of sarcopenia. These elements encompass the participant’s strength level, their requirement for walking assistance, their speed in rising from a chair, their ability to climb stairs, and their history of falls. Each question is scored on a range from 0 to 2, with a higher score indicating more severe sarcopenia symptoms. An overall score above 4 is indicative of sarcopenia. The specificity of the SARC-F questionnaire in detecting sarcopenia has been shown to have moderate-level accuracy [17]. We obtained permission to use the Mandarin Chinese version of the SARC-F questionnaire from Dr. Tai-Yin Wu [18].

### 2.4. Statistics

G power (version 3.1.9.7) was applied to calculate the essential sample size, with set linear bivariate regression, αerror 0.05, power 0.95, and a required total sample size of 238 (119 in each group). Hence, a total of 409 participants is an appropriate sample size in this study. Statistical analysis was conducted to evaluate the relationship between oral frailty and associated factors. All analyses were performed using SPSS software (version 20). Chi-square tests were used to analyze the association between categorical variables. For continuous variables, the Mann–Whitney U Test was used. Logistic regression was applied to examine the association between oral frailty (based on the OFI-8 score) and SARC-F scores, with adjustments for potential confounders, including age, sex, and other relevant factors.

## 3. Results

### 3.1. Demographic Characteristics

As shown in Table 1, among the total 409 participants, 100 males (58.5%) and 114 females (47.9%) had oral frailty. Participants with oral frailty were significantly older than those without oral frailty (*p*-value < 0.001). The BMI of participants with oral frailty was greater than that of those without oral frailty (*p*-value 0.005). Participants with oral frailty had lower education levels than those without oral frailty (*p*-value < 0.001). SARC-F scores were significantly higher in the oral frailty group than in the non-oral frailty group (*p* < 0.001). The results for the OFI-8 showed a significant difference between the two groups, with the non-oral frailty group having a median score of 2 (IOR: 1–3) and the oral frailty group a higher median score of 5 (IOR: 4–7), with *p* < 0.001.

### 3.2. OFI-8 and SARC-F

Table 2 presents the logistic regression model of oral frailty, considering variables such as SARC-F, age, gender, BMI, and education. In the crude model, participants with higher SARC-F scores exhibited a significantly elevated risk of oral frailty, with a crude odds ratio (OR) of 2.114 (95% CI 1.629–2.742, *p*-value < 0.001). Moreover, participants who were older, male, had a higher BMI, and possessed lower education levels also demonstrated a significantly increased risk of oral frailty (crude OR 1.088, 1.532, 1.050, and 3.426, respectively). Furthermore, participants with higher SARC-F scores continued to exhibit a heightened risk of oral frailty even after adjusting for potential confounding factors, with an adjusted odds ratio of 2.130 (95% CI 1.580–2.872, *p*-value < 0.001). After adjusting for comorbidities, the results revealed the same trend (Supplementary Table S2).

## 4. Discussion

This study aims to establish a Mandarin Chinese version of the OFI-8 questionnaire and to explore the relationship between oral frailty and sarcopenia in Taiwan. The hypothesis is that there is a statistically significant correlation between oral frailty and sarcopenia symptoms as assessed through the OFI-8 and SARC-F score. The two different questionnaires were employed to assess the incidence of oral frailty and sarcopenia among 409 elderly Taiwanese participants. The results revealed that 52% of the participants exhibited oral frailty, and there was an association with sarcopenia symptoms, age, gender, and BMI, as well as education level. In the logistic regression analysis of oral frailty, the unadjusted odds ratio indicated that participants with oral frailty had a higher likelihood of experiencing sarcopenia symptoms, having lower education levels, advanced age, and higher BMI scores, and being male. The assessment of oral frailty and sarcopenia was conducted using two questionnaires: the Mandarin Chinese versions of the OFI-8 questionnaire and the SARC-F questionnaire. While the SARC-F questionnaire is a widely recognized international screening method for sarcopenia, the OFI-8 questionnaire was introduced in a 2021 study by Tanaka et al. conducted in Japan [15,19]. The development of the OFI-8 questionnaire originated from a 2017 study by Tanaka et al. which found a strong association between oral frailty, physical frailty, and sarcopenia [20]. In 2021, a new oral frailty measurement questionnaire was generated based on the 2017 study’s findings, providing a more comprehensive assessment of oral frailty [15,20]. The OFI-8 questionnaire consists of eight items related to tooth loss, tooth hygiene frequency, and chewing and swallowing ability, and the results are presented on a scoring system ranging from 0 to 11, where a higher score indicates poorer oral health. The OFI-8 questionnaire covers various aspects of oral muscle condition in more detail. In a UK study that also focused on the effect of poor oral health on physical frailty, the oral health measurement criteria only assessed the number of teeth, periodontal disease, and four self-reported oral health questions; therefore, after a series of considerations, in this study conducted in Taiwan, the intervention applied the OFI-8 questionnaire in Mandarin Chinese to measure oral frailty [21].

In this study, the results demonstrated a robust association between oral frailty and sarcopenia symptoms. This reinforces the notion that oral health influences nutrition intake and the condition of oral muscles, potentially leading to chronic sarcopenia symptoms [2]. These findings align with a 2024 comprehensive review that focused on sarcopenia, dysphagia, malnutrition, and oral frailty. It highlighted that oral frailty limits nutritional intake, including fiber, leading to a higher risk of malnutrition, which is often associated with dysphagia and sarcopenia [5]. Similarly, concerning the factors of education level and age, the two previous Japanese studies also revealed a statistically significant impact of oral frailty among participants with lower education levels and older age [15,20]. A potential explanation of the effect of education can be that it is related to poor health literacy and a lack of understanding of oral health. Two different studies from Finland and Sweden found differences in oral health between participants with different education levels [22,23]. Both studies’ results showed that there was an inequality in the understanding of oral health and that oral health was poorer among participants with a lower education background [22,23]. The factor of age plays a crucial role in both oral health and sarcopenia symptoms. In both this study and Tanaka et al., the results consistently indicate that participants with oral frailty tend to have a higher mean age [20]. This observation can be attributed to the fact that aging individuals may experience tooth loss due to accidents, falls, and diseases.

The risk factors examined in this study align closely with those found in the two Japanese studies [15,20]. However, notable differences emerged, particularly in the results related to BMI and gender. Contrary to expectations, participants with oral frailty in this study exhibited a higher BMI compared to those without oral frailty. Previous research commonly suggests that a lower BMI is strongly associated with oral frailty, indicating poorer nutritional intake and potentially poor oral health [15,20]. This unexpected observation contradicts prevailing beliefs in the literature. While BMI may not decisively influence oral frailty, there is research supporting the association between obesity and poorer oral health. Obesity, defined as a high body fat level, is often linked to insulin resistance, dietary patterns, and body inflammation, all of which can impact oral health and periodontal diseases. In a 2017 observational study comparing obese (>30 BMI) and non-obese (<30 BMI) participants, a strong association between obesity and periodontitis was observed [24]. Another study focusing on the association between high-BMI participants and a periodontitis-related inflammatory index also revealed a correlation [25]. These findings could possibly explain the higher chance of oral frailty among participants with a higher BMI in this intervention. However, this study could not fully confirm the association between high BMI and poorer oral health, as participants did not measure muscle mass, cholesterol level, insulin sensitivity, or inflammation.

Regarding gender-related results, male participants in this study showed a higher odds ratio of oral frailty than their female counterparts. This observation aligns with a US study suggesting disparities in oral health between males and females due to biological and gender-related factors [26]. Male-specific behavior and certain biological reasons have been identified as contributing to poorer oral health, including a higher smoking rate, poorer oral health behavior, and a higher incidence of oral-related diseases [26]. Smoking, in particular, is a significant factor in several oral diseases, such as periodontal and gingival diseases, due to its impact on pathogenic bacteria invasion and the inhibition of the oral immune response [26]. Statistically, US males had a higher smoking rate than females in 2015, and in 2007, males had a lower likelihood of brushing their teeth more than twice a day compared to females, according to the American Dental Association [26]. While research in the US has indicated that male biological factors and oral hygiene behavior contribute to gender disparities in oral health, these underlying factors were not investigated in this study, limiting our ability to affirm these findings [26]. Future research is needed to further explore the relationship between gender and oral frailty.

The strength of this study lies in it being the first research study in Taiwan to focus on measuring both oral frailty and sarcopenia. Additionally, it is the first study to use the OFI-8 questionnaire to measure oral frailty in Taiwan. The findings of this study contribute to the field of oral frailty and sarcopenia research, as the amount of research focusing on the association between these two symptoms is relatively limited to date. Despite some unexpected findings on the risk factors of sarcopenia, this study’s results still make a valuable contribution to the research field of sarcopenia and oral frailty in Taiwan.

### Limitation

The limitations of this study include insufficient measurements of BMI results and gender-related oral health factors. While this study suggests that male participants and those with higher BMI scores have a higher chance of oral frailty, underlying factors such as smoking status, oral hygiene frequency, body muscle mass level, and inflammation levels were not measured, compromising the reliability of these findings. As a result, future confirmation is necessary to establish a potential association with oral frailty. Lastly, due to the cross-sectional study design, this study can only suggest a correlation between oral frailty and sarcopenia, and causation cannot be established.

## 5. Conclusions

This study has successfully developed a practical Mandarin Chinese version of the oral frailty screening tool, the OFI-8. Moreover, our findings indicate a positive association between the risk of oral frailty and the risk of sarcopenia. This assessment tool holds promise for evaluating oral health status in the Chinese community, fostering further research on oral frailty within the Mandarin Chinese-speaking population, and addressing challenges related to defining oral frailty in the future.

## Figures and Tables

**Table 1:** Demographic characteristics of the oral frailty and non-oral frailty groups.

	Non-Oral Frailty (*n* = 195)	Oral Frailty (*n* = 214)	*p*-Value
Gender			0.022 *
Male	71 (41.5%)	100 (58.5%)	
Female	124 (52.1%)	114 (47.9%)	
Age (years)			
Medium (IOR)	65.56 (57.02–69.78)	70.65 (66.36–75.08)	<0.001 *
BMI (kg/m^2^)			
Medium (IQR)	23.62 (21.75–26.16)	25.06 (22.15–27.16)	0.005 *
Education level			<0.001 *
Below high school	46 (29.5%)	110 (70.5%)	
Junior high school and above	149 (58.9%)	104 (41.1%)	
Exercise			0.361
Yes	123 (45.2%)	149 (54.8%)	
No	62 (52.1%)	57 (47.9%)	
Comorbidity			<0.001 *
Yes	73 (37.4%)	133 (62.1%)	
No	122 (62.6%)	81 (37.9%)	
SARC-F			
Medium (IQR)	0 (0–0)	0 (0–2)	<0.001 *
OFI-8			
Medium (IOR)	2 (1–3)	5 (4–7)	<0.001 *

BMI: body mass index; * *p* value < 0.05; Comorbidities: hypertension, diabetes mellitus, hyperlipidemia, cardiovascular disease, pulmonary disease, digestion disease, urology disease, and neurological disease.

**Table 2:** Logistic regression of oral frailty, SARC-F, and potential confounders.

	Crude OR	95% LCI	95% UCI	*p*-Value	Adjusted OR	95% LCI	95% UCI	*p*-Value
SARC-F	2.114	1.629	2.742	<0.001 *	2.130	1.580	2.872	<0.001 *
Age (year)	1.088	1.061	1.115	<0.001 *	1.071	1.041	1.102	<0.001 *
Gender (female)	1.532	1.031	2.277	0.035 *	2.378	1.463	3.865	<0.001 *
BMI (kg/m^2^)	1.050	1.000	1.101	0.048 *	1.055	0.998	1.116	0.059
Education (high)	3.426	2.238	5.244	<0.001 *	1.796	1.050	3.071	0.032 *

OR: odds ratio; LCI: lower confidence interval; UCI: upper confidence interval; * *p* value < 0.05.

## Data Availability

The data that support the findings of this study are available from the corresponding author upon reasonable request.

## Supplementary Materials

The following supporting information can be downloaded at https://www.mdpi.com/article/10.3390/geriatrics10020047/s1, Table S1: Oral frailty index; Table S2: Logistic regression of oral frailty, SARC-F and potential confounders.
