# Supplementary material for: Establishment of a Mandarin Chinese Version of the Oral Frailty Index-8 and Exploration of the Association Between Oral Frailty and Sarcopenia
Source: Geriatrics (Basel). 2025 Mar 17;10(2):47. doi: 10.3390/geriatrics10020047 (PMC11932261; doi:10.3390/geriatrics10020047)
Supplement: Supplementary file 1 [file geriatrics-10-00047-s001.zip › geriatrics-3203089-supplementary.pdf]

Supplementary Materials

Table S1. 口腔衰弱指數 (Oral frailty index).

	是	否
(1)與 6 個月前相比，你在吃堅硬的食物有困難嗎？	□[2]	□[0]
(2)你最近有被茶或湯嗆到嗎？	□[2]	□[0]
(3)你有用假牙嗎？*	□[2]	□[0]
(4)你經常口乾舌燥嗎？	□[1]	□[0]
(5)你出門的頻率比去年少嗎？	□[1]	□[0]
(6)你能吃魷魚乾或醃蘿蔔之類堅硬的食物嗎？	□[0]	□[1]
(7)你一天刷幾次牙？(每天 2 次或更多次)	□[0]	□[1]
(8)您是否至少每年看一次牙科？	□[0]	□[1]

*如果您失去一顆牙齒，使用假牙治療是重要的，這樣您就可以吃堅硬的食物。

Translator: Chen-Cheng Yang. abcmacoto@gmail.com.tw

Table S2. Logistic regression of oral frailty, SARC-F and potential confounders.

	Crude OR	95%LCI	95%UCI	*p*-Value	Adjusted OR	95%LCI	95%UCI	*p*-Value
SARC-F	2.114	1.629	2.742	<0.001*	2.074	1.536	2.801	<0.001*
Age (year)	1.088	1.061	1.115	<0.001*	1.060	1.027	1.093	<0.001*
Gender (female)	1.532	1.031	2.277	0.035*	2.494	1.508	4.123	<0.001*
BMI (kg/m^2)	1.050	1.000	1.101	0.048*	1.048	0.990	1.109	0.106
Education (high)	3.426	2.238	5.244	<0.001*	0.813	0.678	0.975	0.025*
Comorbidity (ref: no)	2.744	1.838	4.096	<0.001*	1.637	1.025	2.614	0.039*

OR: odds ratio; LCI: lower confidence interval; UCI: upper confidence interval; * p value < 0.05.
